# Agreement between transperineal ultrasound measurements and digital examinations of cervical dilatation during labor

**DOI:** 10.1186/s12884-015-0704-z

**Published:** 2015-10-24

**Authors:** Sigurlaug Benediktsdottir, Torbjørn M. Eggebø, Kjell Å. Salvesen

**Affiliations:** Department of Obstetrics and Gynecology, Clinical Sciences, Lund University, Lund, Sweden; National Center for Fetal Medicine, Trondheim University Hospital (St Olavs Hospital), Trondheim, Norway; Department of Laboratory Medicine, Children’s and Women’s Health, Norwegian University of Science and Technology, Trondheim, Norway

## Abstract

**Background:**

To compare 2D transperineal ultrasound assessment of cervical dilatation with vaginal examination and to investigate intra-observer variability of the ultrasound method.

**Methods:**

A prospective observational study was performed at Skane University Hospital, Lund, Sweden between October 2013 and June 2014. Women with one fetus in cephalic presentation at term had the cervical dilatation assessed with ultrasound and digital vaginal examinations during labor. Inter-method agreement between ultrasound and digital examinations and intra-observer repeatability of ultrasound examinations were tested.

**Results:**

Cervical dilatation was successfully assessed with ultrasound in 61/86 (71 %) women. The mean difference between cervical dilatation and ultrasound measurement was 0.9 cm (95 % CI 0.47–1.34). Interclass correlation coefficient (ICC) was 0.83 (95 % CI 0.72–0.90). Intra-observer repeatability was analysed in 26 women. The intra-observer ICC was 0.99 (95 % CI 0.97–0.99). The repeatability coefficient was ± 0.68 (95 % CI 0.45–0.91).

**Conclusion:**

The mean ultrasound measurement of cervical dilatation was approximately 1 cm less than clinical assessment. The intra-observer repeatability of ultrasound measurements was high.

## Background

Labor management is based on clinical evaluation of cervical dilatation and descent and rotation of the presenting part. Digital vaginal examination (VE) is highly subjective and operator dependent [1–4]. Some women experience VE as intimidating and uncomfortable, and repeated VEs can increase the risk of infection [5].

In recent years intrapartum sonography has been used as a complement to traditional clinical examinations. Examination of viability, fetal lie, presentation and position of the head [6–8] can be done by transabdominal ultrasound. Fetal station can be assessed with a transperineal approach by measuring fetal head-perineum distance [9–12] or angle of progression [13, 14]. Women report less pain when examined with transperineal ultrasound compared to digital examination [15].

An objective painless and simple method for assessment of cervical dilatation is warranted. Zimerman et al. has published a 3D ultrasound method [16], and Hassan et al. has suggested how to examine cervical dilatation in 2D [17]. The aims of the present study were to compare 2D ultrasound assessment of cervical dilatation with vaginal examination and to investigate intra-observer variability of the ultrasound method.

## Methods

We performed a prospective observational study among 86 women in Lund, Sweden between October 2013 and June 2014. Women were recruited when a member of the study team was available, and all women gave written informed consent to participate. The Local Ethical Review Board (Lund, Sweden) approved the study (Diary number 2013/470).

**Fig. 1:**
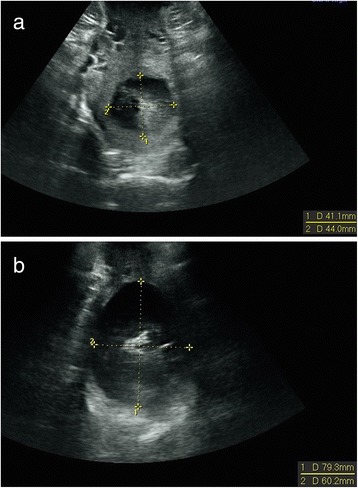
Transperineal (2D) ultrasound measurement of cervical dilatation at (a) 4,3 cm and (b) 7,0 cm

Women with cephalic presentation at ≥37 weeks of gestation were eligible for the study. Women in all stages of labor were examined while in a supine position with flexed knees and hips and with an empty bladder. Acquisitions were performed between contractions with a Voluson *i* ultrasound machine (GE Medical Systems, Zipf, Austria) equipped with a 3.5–7.5 MHz 3D curved multifrequency transabdominal transducer. The transducer was covered with a glove and placed between labia majora in the posterior fourchette. The cervical dilatation was measured in the transverse view as described by Hassan et al. [17]. We used the mean value of the anterior-posterior and transverse diameters with the cursors placed on the inside of the cervix (inner-to-inner) as seen in Fig. 1. Two doctors and two midwives did the ultrasound examinations and 33 attending midwives did the digital examinations. The ultrasound operators were not involved in the clinical management of labor, and ultrasound operators and attending midwives were blinded to each other’s assessments.

### Statistical analyses

The analysis of inter-method agreement was performed using the mean of three ultrasound measurements and one digital assessment. If zero was inside the 95 % CI of the difference, no bias was assumed. To assess systematic bias between ultrasound measurements and digital palpation, differences between values were plotted against means of the measurements. Limits of agreement with 95 % CIs of the lower and upper limits were calculated as described by Bland and Altman [18]. Inter-method agreement was also expressed using intraclass correlation coefficient (ICC) calculated as two-way random variation of average measurements. Linear regression analysis was performed to investigate the association between ultrasound measurements of the cervix and digital palpation. Correlations were expressed using the Pearson correlation coefficient (r).

Intra-observer repeatability of the measurements was expressed as the difference between the highest and lowest measurements and the repeatability coefficient. The differences between the first, second and third measurements were evaluated with three-way analysis of variance, and intra-observer ICC was calculated using two-way random variation of single measurements.

The data were analysed with the statistical software package SPSS statistics version 21.0 (IBM SPSS, Armonk, NY, USA).

## Results

**Fig. 2:**
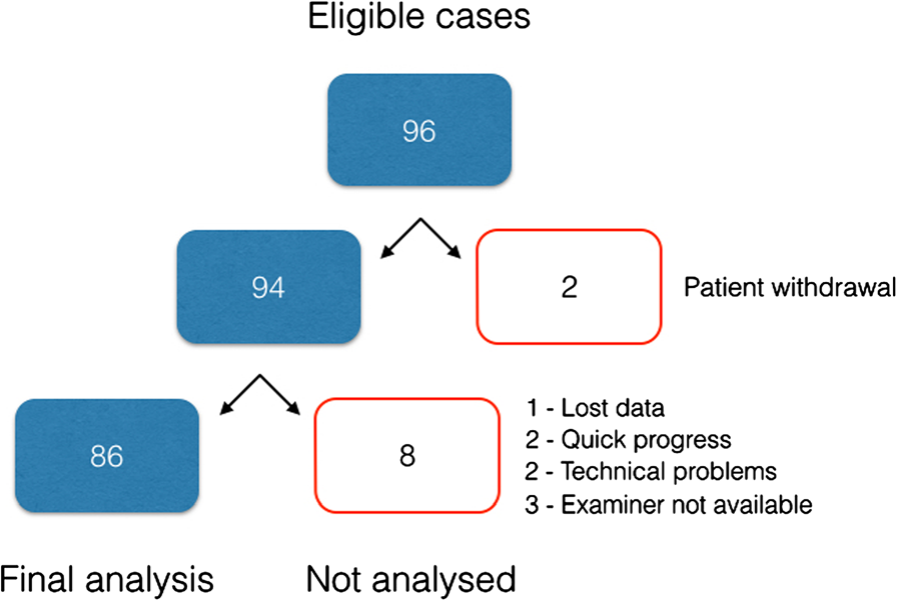
Flow-chart illustrating the study population

In all, 96 women were eligible for the study, and 86 women were included in the analysis. Details are presented as a flow-chart in Fig. 2.

Maternal characteristics are presented in Table 1. Sixty-four (74 %) of 86 women were in active labor defined as cervix being dilated ≥4 cm. The remaining women (*n* = 22) were in the latent phase.

**Table 1:** Characteristics of the study population

Characteristics	*n* = 86 median (range) or *n* (%)
Mother	
Maternal age (years)	30.5 (23–43)
Body mass index (kg/m^2^)	24.3 (18–36)
Gestational age (weeks)	40 (36–42)
Parity	1 (0–5)
Labor	
Induction of labor	23 (27)
Augmentation of labor	56 (65)
Epidural analgesia	38 (44)
Cesarean delivery	7 (8)
Operative vaginal delivery	13 (15)
Newborn	
Birth weight (g)	3665 (2010–4780)

Cervical dilatation was successfully assessed with ultrasound in 61/86 (71 %) women, and there was missing data from palpation in 2 women. More than half of 25 missing cases in the ultrasound group were found when cervical dilatation was ≥8 cm; 8/25 (32 %) was fully dilated and 5/25 (20 %) was 8–9 cm dilated. When cervix was palpated ≥8cm dilated, we were unable to measure cervical dilatation with ultrasound in 65 % of women (13/20).

**Fig. 3:**
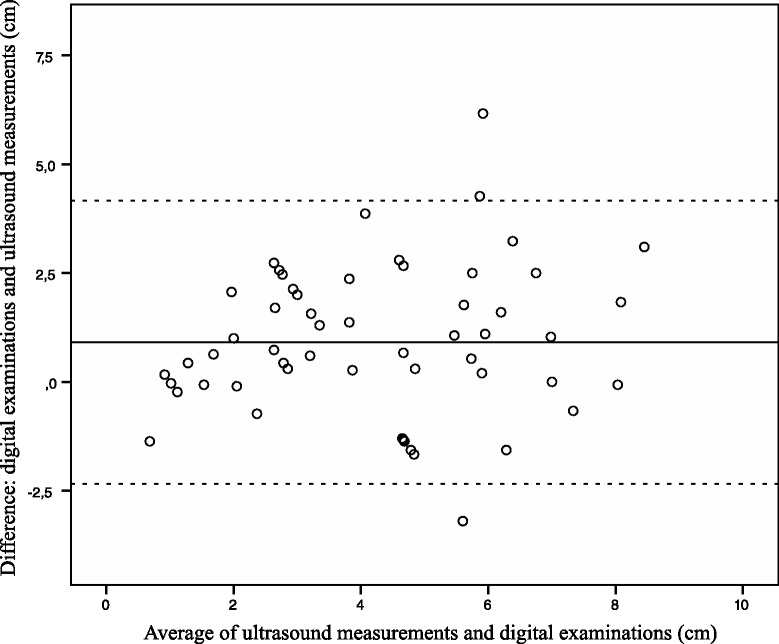
Bland-Altman plot of intermethod agreement between digital examinations and ultrasound measurements of cervical dilatation. Mean difference and limits of agreement

Ultrasound measurements and clinical assessments were compared in 59 women. The mean cervical dilatation measured with ultrasound was 3.8 cm, median 3.3, (range 0.8–8.1) and the mean cervical dilatation with palpation was 4.7 cm, median 4.0, (range 0–10). The mean difference between cervical dilatation and ultrasound measurement was 0.9 cm (95 % CI 0.47–1.34). ICC was 0.83 (95 % CI 0.72–0.90). The agreement between the methods is presented as a Bland-Altman plot (Fig. 3). Limits of agreement were −2.34 to 4.16. Details are presented in Table 2.

**Table 2:** Intermethod agreement between ultrasound examinations and digital palpations

Cervix dilatation (cm)				Difference between the 2 methods (cm)							
Mean	Median	Range	Inter-CC (95 % CI)	Mean	95 % CI of mean	1.96 SD	Lower limit	Upper limit	95 % CI of lower limit	95 % CI of upper limit	Range
4.24	4.65	0.68 to 8.45	0.83 (0.72–0.90)	0.91	0.47 to 1.34	3.25	−2.34	4.16	−3.07 to −1.61	3.43 to 4.89	−3.2 to 6.17

Mean, median and range for cervix dilatation are calculated from the mean of the 2 methods

*Inter-CC* interclass correlation coefficient, *SD* standard deviation

**Fig. 4:**
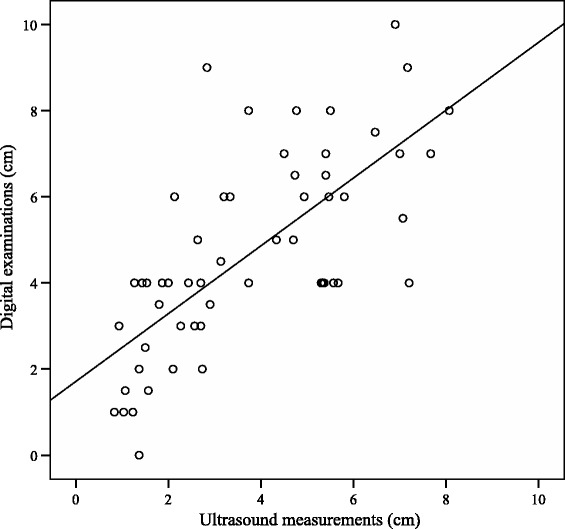
Scatter plot illustrating the association between ultrasound measurements and digital examinations of cervical dilatation

The association between ultrasound measurements and digital examinations is presented in Fig. 4. The regression equation was y = 1.7 + 0.8x. Pearson correlation coefficient was 0.72 (95 % CI 0.56–0.82).

One examiner (SB) did 40 ultrasound examinations in which three measurements were successfully obtained in 26 women. The mean dilatation was 4.63cm in the first, 4.51cm in the second and 4.45cm in the third measurements. This was a significant trend (*p* = 0.03). The intra-observer ICC was 0.99 (95 % CI 0.97–0.99) and the repeatability coefficient was ± 0.68 (95 % CI 0.45–0.91)). Details are given in Table 3.

**Table 3:** Intraobserver repetability for ultrasound measurements of cervical dilatation

Cervical dilatation (cm)					Difference between highest and lowest values (cm)				
Mean	Median	Range	Intra-CC (95%CI)	Repeatability coefficient (cm) (95%CI)	Mean	Median	10th centile	90th centile	Range
4.53	4.60	1.37–7.20	0.99 (0.97–0.99)	±0.68 (0.45–0.91)	0.38	0.30	0.10	0.70	0–1.0

Mean, median and range of ultrasound examinations of cervical dilatation are calculated from the mean of 3 measurements

*Intra-CC* intraclass correlation coefficient

## Discussion

We found transperineal ultrasound to be a suitable method to assess cervical dilatation during first stage of labor. When cervix was ≥8cm dilated, we were unable to measure cervical dilatation in 65 % of women. Ultrasound measurement of cervical dilatation was on average 1 cm less than digital assessment. Intra-observer repeatability of the ultrasound method was very good with ICC 0.99.

Earlier studies comparing agreement of digital assessment of cervical dilatation have shown inconsistent results. In two previous studies, in which two examiners performed VE during labor, complete agreement of cervical dilatation was found in 42–49 % of cases, and 90 % agreement was observed if 1 cm difference was allowed [4, 19]. Another study used a spatial position-tracking ruler attached to the examiners fingertips. That study found an overall examination accuracy of ≤1cm in 53 % of women with mean error 10.2 ± 8.4 mm [20]. Both Nizard et al. [20] and Buchman et al. [4] found that the accuracy of VE is best at the lower (1–4 cm) and upper end (>8 cm) of the scale for cervical dilatation. When cervix was fully dilated, the accuracy was around 75 %. When the cervical dilatation was 6–8cm, the accuracy of VE was 36–38 % [4, 20]. In vitro studies on models confirm this [21, 22]. Phelps et al. found that the overall accuracy was 56 %, however, with 1 cm error margin the accuracy improved to 90 % [22]. In vitro study with soft models have poorer accuracies (19 %) [21].

Publications on ultrasound measurement of cervical dilatation during labor are sparse. Yuce et al. found that the agreement between VE and ultrasound measurement of cervical dilatation was good with ICC 0.82 (95 % CI 0.73–0.88), and that ultrasound measures the cervical diameter 10 mm smaller compared to VE [23]. Zimerman et al. [16] described how to measure cervical dilatation offline with transperineal 3D ultrasound technique and found that the mean cervical diameter had good correlation with VE. Hassan et al. [17] used 2D ultrasound measurements of the anterior-posterior diameter and found that the mean absolute difference was 1.24 cm between ultrasound measurement and VE. It is suggested that VE consistently overestimates the degree of cervical dilatation compared to ultrasound [24].

There are some limitations of the present study. We aimed to perform ultrasound and clinical examinations within a short time span but we did not register the time interval. We excluded two cases from the analyses because of very quick deliveries, but we cannot rule out a possibility that the observed differences were due to progression during the time interval. New studies adjusting for time intervals between examinations should be done.

Another limitation was that we did not register rupture of the membranes. In retrospective experience, it is easier to measure the cervix with ultrasound when the membranes are intact. Future studies must examine if ultrasound performs better in a group of women with intact membranes. A third limitation was that ultrasound were performed by four operators whereas digital palpations were done by 33 midwives. However, we will argue that this reflects everyday clinical practice in a busy labor ward.

Transperineal ultrasound measured the cervix 9 mm less dilated compared to digital palpation. This difference can be explained by the fact that cervix will distend when the examiner inserts the fingers into the cervical canal. We performed a transverse scan and measured cervical dilatation as the mean value of the anterior-posterior and the transverse diameters. Hassan et al. [17] used the anterior-posterior diameter alone in their study. It can be argued from ultrasound physics that the best measurements are obtained in the measurement plane of the anterior-posterior diameter. However, we will argue that the mean of two diameters is more appropriate when comparing with digital palpation because the examiner usually will spread the fingers in the horizontal plane. An intraobserver analysis from one operator demonstrated that the mean dilatation of cervix measured by ultrasound, decreased between the first, second and third ultrasound measurement from 4.63 cm in the first to 4.45 cm in the last examination. This difference was statistically significant, but not clinically important. The trend was not significant including analyses from all examiners (*p* = 0.21). The study population was too small to perform separate intraobserver analyses of all examiners.

We were unable to measure cervical dilatation in 65 % of the women in late first stage and during second stage of labor. Measurements during these stages are more difficult because shadowing from the fetal skull makes it more difficult to identify the cervix as a distinct ring and because a thin cervix is more difficult to visualise.

Transperineal ultrasound can be used as a complement to traditional clinical examinations. Fetal station and position is more accurately assessed with ultrasound [8, 25, 26], and the sonopartogram has been launched as a possible replacement of the traditional partogram [24]. Women report less pain when examined with transperineal ultrasound compared to digital examination [15] and replacing some of the clinical examinations with transperineal ultrasound examinations might decrease the risk of infection. Longitudinal studies evaluating the sonopartogram in normal and prolonged labor are needed.

## Conclusions

In conclusion, we found that transperineal ultrasound is a suitable method to assess cervical dilatation in latent and early active stages of labor.

## Abbreviations

ICC: Intraclass correlation coefficient
VE: Vaginal examination
2D: Two dimensional
3D: Three dimensional
